# Supplementary material for: Development of a Simple Kinetic Mathematical Model of Aggregation of Particles or Clustering of Receptors
Source: Life (Basel). 2020 Jun 26;10(6):97. doi: 10.3390/life10060097 (PMC7345685; doi:10.3390/life10060097)
Supplement: Supplementary file 1 [file life-10-00097-s001.pdf]

Supporting information

Development of a simple kinetic mathematical model of aggregation of particles or clustering of receptors

Andrei K. Garzon Dasgupta[1,2], Alexey A. Martyanov[1,2,3,4], Aleksandra A. Filkova[1,2,4], Mikhail A. Panteleev[1,2,4,5] and Anastasia N. Sveshnikova[1,2,4,6,*]

1 Faculty of Physics, Lomonosov Moscow State University, 1/2 Leninskie gory, Moscow, 119991, Russia
2 Center for Theoretical Problems of Physico-Chemical Pharmacology, Russian Academy of Sciences, 30 Srednyaya Kalitnikovskaya str., Moscow, 109029, Russia
3 Institute for Biochemical Physics (IBCP), Russian Academy of Sciences (RAS), Russian Federation, Moscow, Kosyigina 4; 119334, Russia
4 National Medical Research Center of Pediatric Hematology, Oncology and Immunology named after Dmitry Rogachev, 1 Samory Mashela St, Moscow, 117198, Russia
5 Faculty of Biological and Medical Physics, Moscow Institute of Physics and Technology, 9 Institutskii per., Dolgoprudnyi, 141700, Russia
6 Department of Normal Physiology, Sechenov First Moscow State Medical University, 8/2 Trubetskaya St., Moscow, 119991, Russia
* Correspondence: a.sveshnikova@physics.msu.ru

Table S1. Automatically assessed model parameters for experimental datasets given on Fig. 3a

Parameter	Simple model		Parameter	Aggregation model
k_{-1}	$1.0*10^{-4}$		k_{-1}	$4.5*10^{-17}$
k_1	0.018		k_1	0.041
k_{-2}	$8.13*10^{-5}$		k_{-2}	$3.88*10^{-16}$
k_2	$8.05*10^{-13}$		k_2	$6.3*10^{-3}$
k_3	$0.92*10^{-3}$		k_3	$4.9*10^{-3}$
r_0	2.00		r_0	1.01

Table S2. Automatically assessed model parameters for experimental datasets given on Fig. 4b

Parameter	Simple model		Parameter	Clustering model
k_{-1}	$3.54*10^{-6}$		k_{-1}	$2.7*10^{-5}$
k_1	$1.14*10^{-3}$		k_1	0.95
k_{-2}	$2.64*10^{-8}$		k_{-2}	1.32
k_2	$6.32*10^{-8}$		k_2	$4.6*10^{-4}$
k_3	$1.91*10^{-3}$			
r_0	10000		r_0	10000

Table S3. Model comparisons based on the Akaike's Information Criterion (AIC).

Model	K	n		RSS		AIC	
		2uM ADP	3uM ADP	2uM ADP	3uM ADP	2uM ADP	3uM ADP

Aggregation	7	497	497	0,028	0,069	-1764	-1317
Simple (for aggregation)	7	497	497	0,029	0,033	-1740	-1686
		Col-III	III-30	Col-III	III-30	Col-III	III-30
Clustering	5	52	39	89,2	54,3	243,5	165,8
Simple (for clustering)	6	52	39	87,7	55,0	244,6	168,3

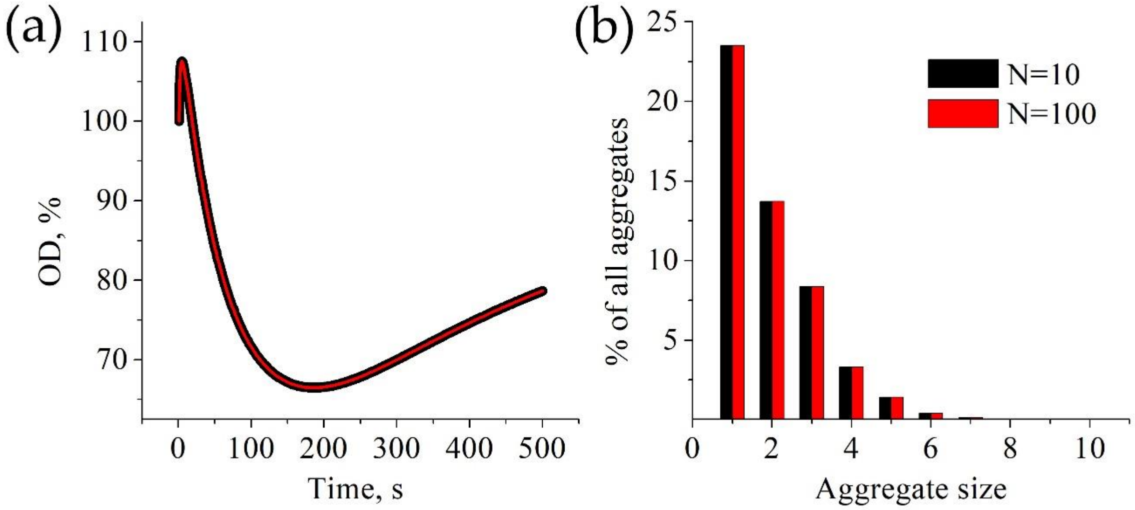

Figure S1. Comparison of size restrictions on "Aggregation model". The impact of the maximal size restriction (N) of "Aggregation" model on the resulting parameters for one set of experimental data. N does not affect OD and other kinetic curves (for N>10). (A) OD-curve for set of parameters presented in Fig. 1E for N=10 (black) and N=100 (red). (B) Distribution of aggregates by their size at timepoint t=200s. For N=100 only aggregates with size 1-10 are represented.

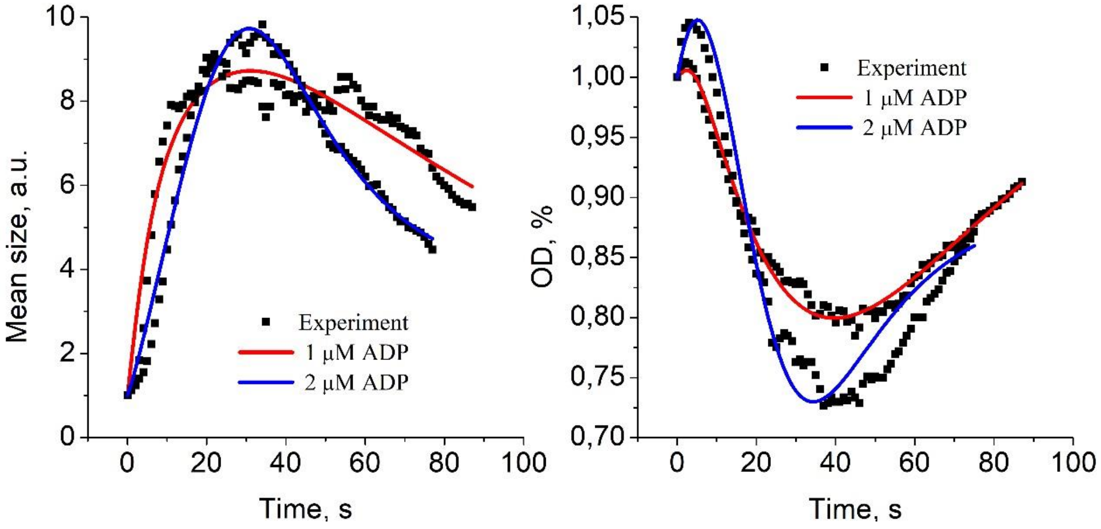

Figure S2. Parameter estimation for "Aggregation model" based on data of optical density and mean aggregate size in platelet aggregation assay. Estimation of model parameters was conducted automatically. The resulting parameters are presented in Table S4.

Table S4. Automatically assessed model parameters for experimental datasets given on Fig. S2

Parameter	1μM of ADP		Parameter	2μM of ADP
k_{-1}	$1.0*10^{-17}$		k_{-1}	$4.6*10^{-19}$
k_1	$1.6*10^{-6}$		k_1	$2.1*10^{-6}$
k_{-2}	$8.6*10^{-17}$		k_{-2}	$4.7*10^{-8}$
k_2	$3.4*10^{-7}$		k_2	$7.4*10^{-8}$
k_3	0.017		k_3	0.05
r_0	146244		r_0	166676

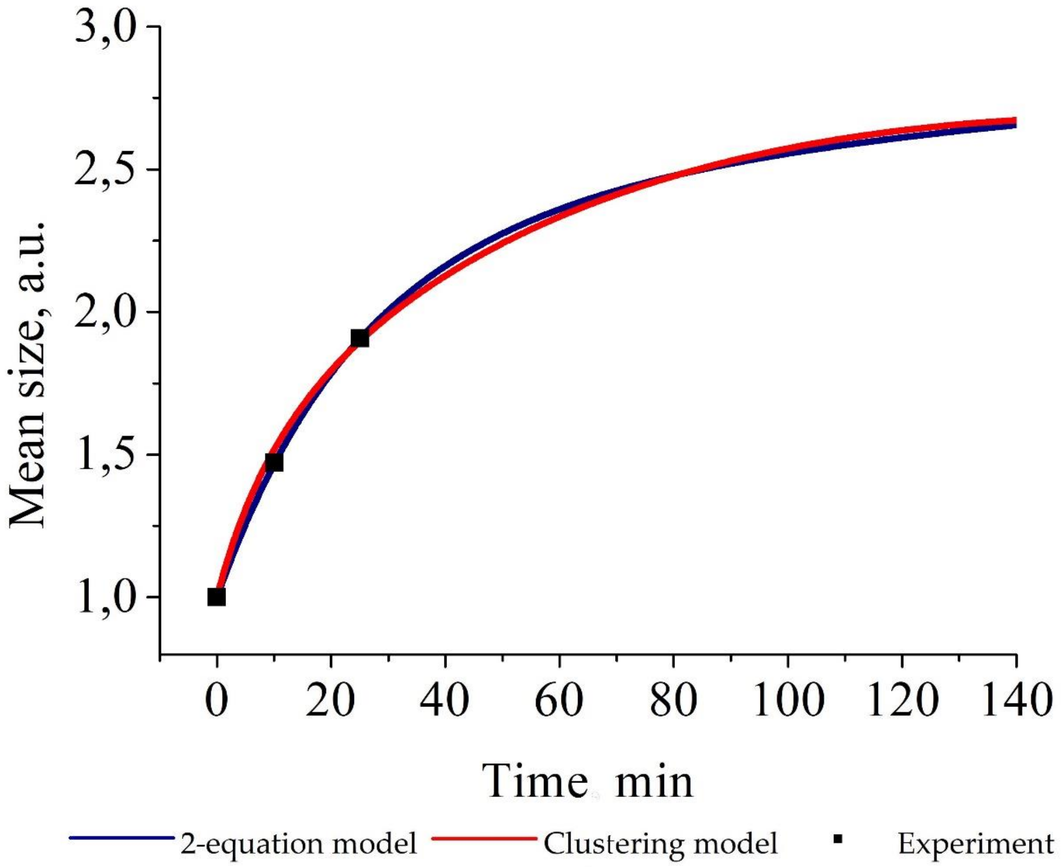

Figure S3. Parameter estimation for "Clustering model" and "2-equation model" based on neutrophil CR3 receptor clustering. Estimation of model parameters for each model and initial concentration was conducted automatically. For each set of experimental data, parameters of the models were estimated independently. Receptor clustering was induced by phorbol myristate acetate (PMA).

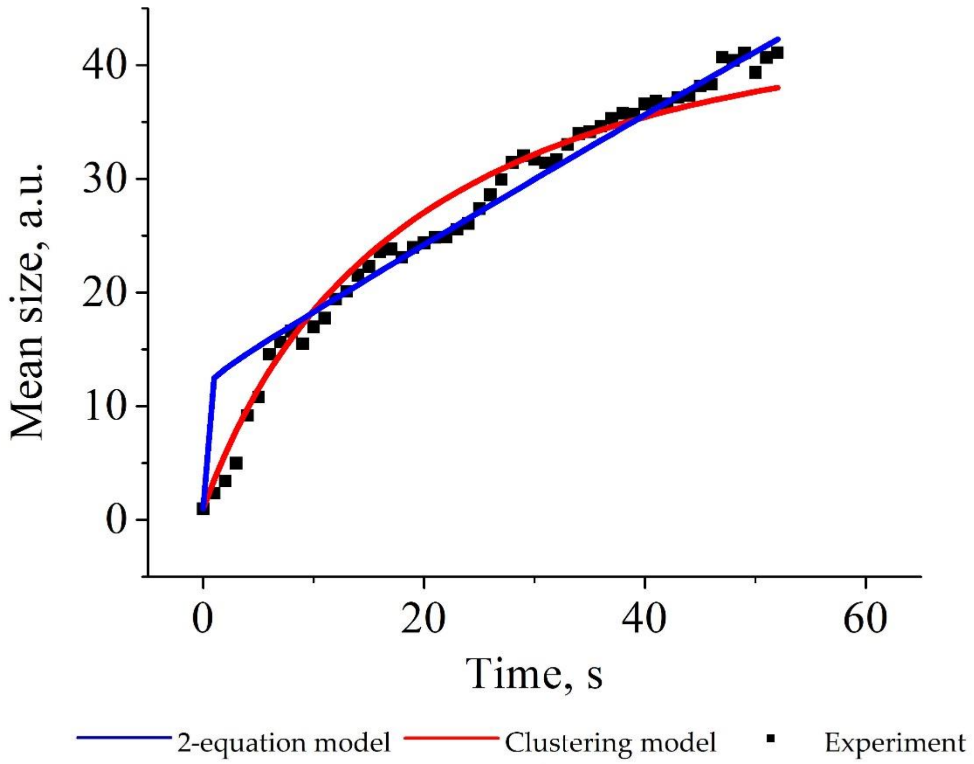

Figure S4. Parameter estimation for "Clustering model" and "2-equation model" based on platelet GPVI receptor clustering with additional restrictions. Estimation of model parameters for each model was conducted automatically. The resulting parameters for "Clustering model": k_1 = $4.2*10^{-3}$, k_{-1} = $1.0*10^{-4}$, k_2 = $2.1*10^{-3}$, k_{-2} = $5.1*10^{-5}$, p_0 = 62,0. For "2-equation model": k_1 = $4.2*10^{-3}$, k_{-1} = $1.0*10^{-4}$, k_{-2} = $2.1*10^{-3}$, k_2 = $4.2*10^{-3}$, k_3 = 0, p_0 = 62,0

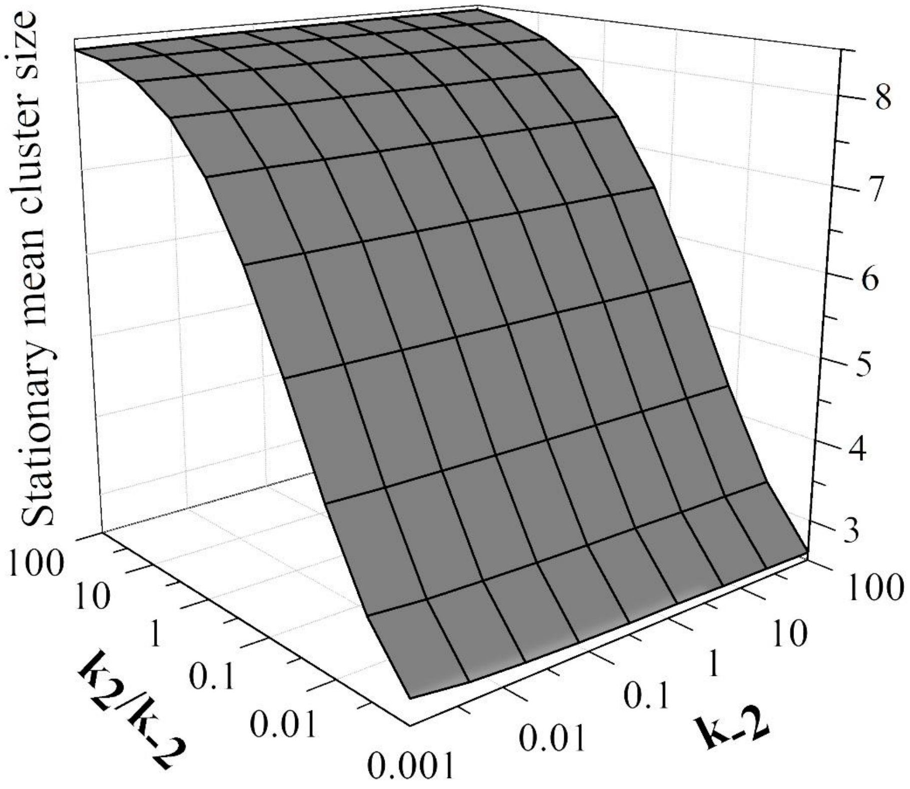

Figure S5. "Clustering model" steady state. The stationary state for the most part is governed by ratios k2/k-2, k1/k-1 that represent Ka of the corresponding reactions. Stationary mean cluster size has a weak dependence on k-2 by itself. Plot is given for fixed k1=1 and k-1=1 values, size restriction N=10.

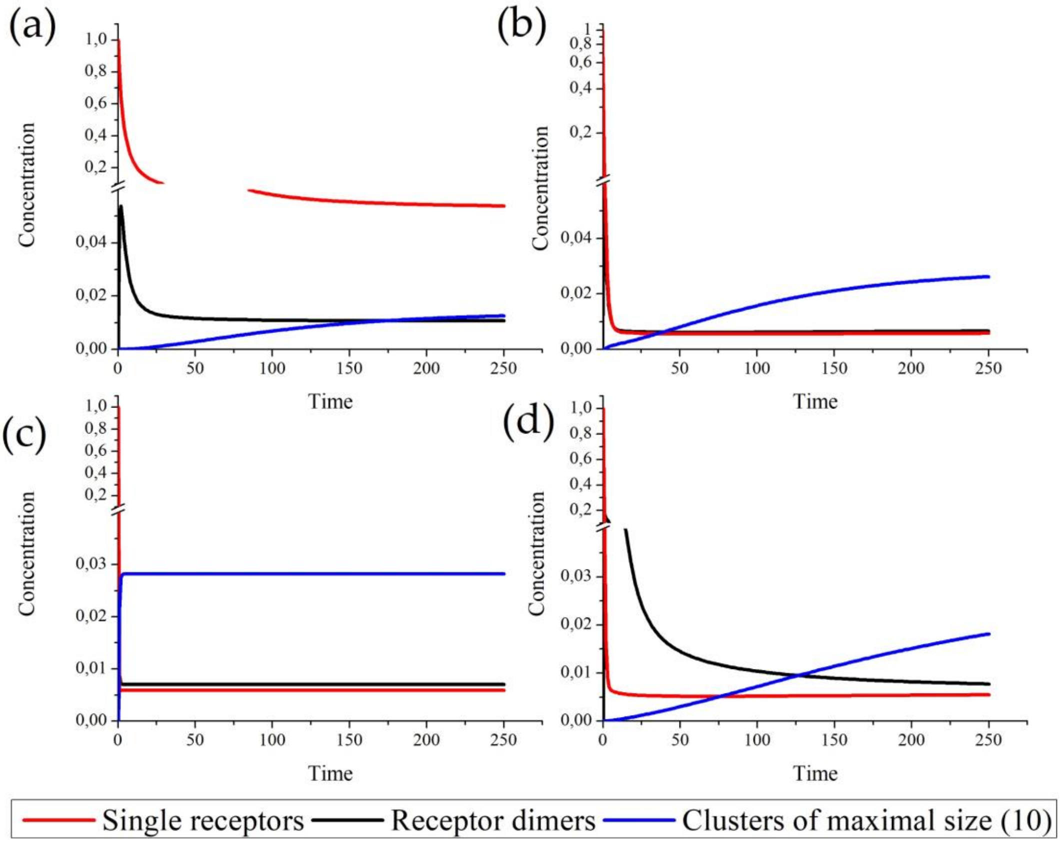

Figure S6. Kinetics of different receptors in "Clustering model". Representation of two types of steady state. The parameters k_{-1}=0.01, k_{-2}=0.01, p_o=1 and N=10 were fixed for (a) and (b). (a)Unclustered steady state in case k1/k-1=10, k2/k-2=100, (b-d) Fully clustered in case of k1/k-1=200, k2/k-2=200, (c) k_{-1}=10, (d) k_{-2}=0.001.
